# Healthcare costs in women with metastatic breast cancer receiving chemotherapy as their principal treatment modality

**DOI:** 10.1186/1471-2407-11-250

**Published:** 2011-06-15

**Authors:** Montserrat Vera-Llonch, Derek Weycker, Andrew Glass, Sue Gao, Rohit Borker, Angie Qin, Gerry Oster

**Affiliations:** 1 Policy Analysis Inc (PAI), Brookline, MA, USA; 2 Kaiser Permanente Northwest, Portland, OR, USA; 3 Amgen Inc, Thousand Oaks, CA, USA

**Keywords:** Cost, metastatic breast cancer, chemotherapy, burden of illness

## Abstract

**Background:**

The economic costs of treating patients with metastatic breast cancer have been examined in several studies, but available estimates of economic burden are at least a decade old. In this study, we characterize healthcare utilization and costs in the US among women with metastatic breast cancer receiving chemotherapy as their principal treatment modality.

**Methods:**

Using a large private health insurance claims database (2000-2006), we identified all women initiating chemotherapy for metastatic breast cancer with no evidence of receipt of concomitant or subsequent hormonal therapy, or receipt of trastuzumab at anytime. Healthcare utilization and costs (inpatient, outpatient, medication) were estimated on a cumulative basis from date of chemotherapy initiation ("index date") to date of disenrollment from the health plan or the end of the study period, whichever occurred first. Study measures were cumulated over time using the Kaplan-Meier Sample Average (KMSA) method; 95% CIs were generated using nonparametric bootstrapping. Findings also were examined among the subgroup of patients with uncensored data.

**Results:**

The study population consisted of 1444 women; mean (SD) age was 59.1 (12.1) years. Over a mean follow-up of 532 days (range: 3 to 2412), study subjects averaged 1.7 hospital admissions, 10.7 inpatient days, and 83.6 physician office and hospital outpatient visits. Mean (95% CI) cumulative total healthcare costs were $128,556 ($118,409, $137,644) per patient. Outpatient services accounted for 29% of total costs, followed by medication other than chemotherapy (26%), chemotherapy (25%), and inpatient care (20%).

**Conclusions:**

Healthcare costs-especially in the outpatient setting--are substantial among women with metastatic breast cancer for whom treatment options other than chemotherapy are limited.

## Background

Breast cancer is the most common form of cancer among women in the US, and the second leading cause of cancer death [1]. It is estimated that one in every eight women will develop breast cancer during their lifetime. In 2009, an estimated 192,370 women were diagnosed with breast cancer, and 40,170 women died from the disease [1]. Approximately 6% of women with incident breast cancer have metastatic disease at initial presentation. An additional 20-40% of breast cancer patients develop metastatic disease at some point following diagnosis. Median survival in women with metastatic breast cancer is about 18-24 months [2], but many patients survive several years. Breast cancer is a heterogeneous disease that is managed with a range of treatment modalities. Approximately two-thirds of breast cancer tumors are hormone-receptor positive (i.e., express estrogen and/or progesterone receptors) [3], and endocrine therapy is considered for these patients. Between 20% and 30% of patients with breast cancer have tumors that express HER-2/neu (HER-2), a tyrosine kinase growth factor receptor located on cell membranes [4]. Targeted therapy with the monoclonal antibody, trastuzumab (Herceptin^®^), or the dual tyrosine kinase inhibitor, lapatinib (Tykerb^®^), in combination with hormonal and/or conventional chemotherapy, has been reported to improve response rates in these patients [5,6]. For patients with tumors that do not express hormone receptors and those with HER-2 negative tumors, chemotherapy remains the main treatment option [7]. Chemotherapy also is the primary treatment modality for patients with rapidly progressive visceral disease and those with hormone-receptor positive tumors that do not respond or have become resistant to endocrine therapy [8,9]. Multiple retrospective studies [10-12] have reported that prognosis is poor among these patients, which could result from the lack of therapeutic options or inherent tumor aggressiveness [9]. Such findings underscore the high levels of unmet clinical need and poor outcomes in this particular subset of patients.

The economic costs of treating women with metastatic breast cancer have been examined in several studies, but none to the best of our knowledge has reported costs for patient subgroups defined on the basis of tumor receptor expression and/or treatment modality. Moreover, available estimates of the economic burden of metastatic breast cancer are at least a decade old. While a number of recent studies have evaluated the economic burden of breast cancer, including all women with this disease irrespective of stage, they did not attempt to characterize disease burden separately for those with metastatic versus earlier-stage disease [13-15]. Disease stage may be a particularly important consideration when evaluating the economic burden of breast cancer as diagnosis, treatment, and follow-up--and the costs thereof--would be expected to vary by stage. In this study, we examine costs in women with metastatic breast cancer receiving chemotherapy as their principal treatment modality.

## Methods

### Data Source

Data for this study were obtained from a large private health insurance claims database (Thomson Reuters Marketscan Research Databases), and spanned the period January 1, 2000 through December 31, 2006. The database is comprised of medical (i.e., facility and professional service) and outpatient pharmacy claims from employer-sponsored health insurance plans covering more than 10 million persons annually, including employees as well as their spouses and dependents. The plans provide health benefits under a number of different products, including fee-for-service and capitated (full, partial) systems. Plan members reside throughout the US; approximately 10% are aged 65 years or older.

Data available for each facility and professional-service claim include date and place of service, diagnoses (in International Classification of Diseases, Ninth Edition, Clinical Modification [ICD-9-CM] format), procedures performed/services rendered (in Health Care Financing Administration Common Procedure Coding System [HCPCS], ICD-9-CM, and Uniform Bill-92 [UB-92] formats), and quantity of services (professional-service claims only). Data available for each retail pharmacy claim include the drug dispensed (in National Drug Code [NDC] format), dispensing date, quantity dispensed, and number of days of therapy supplied. All claims include paid (i.e., reimbursed) amounts, including patient deductibles, copays, and/or coinsurance amounts; for hospital facility claims, paid amounts include all services (including drugs) provided by the institution during the hospital stay. Selected demographic and eligibility information is also available for persons in the database, including age, sex, geographic location, coverage type, and the start and end dates of health insurance coverage. Patient-level data can be arrayed chronologically to provide a detailed longitudinal profile of all medical and pharmacy services received.

All patient-identifying information is either fully encrypted or removed, and the database is therefore compliant with the Health Insurance Portability and Accountability Act of 1996 and federal guidance on Public Welfare and the Protection of Human Subjects. Per the Code of Federal Regulations (45 CFR 46 §46.101), IRB review was not needed for a study of this nature, since "... subjects cannot be identified, directly or through identifiers linked to the subjects..." We had full authorization to use the study database and full access thereto for purposes of the research described herein.

### Study Subjects

The study population consisted of all women, aged 18 years or older, who initiated chemotherapy for metastatic breast cancer and had no evidence of concomitant or subsequent receipt of hormonal therapy, or receipt of trastuzumab at anytime (to limit attention to patients with HER-2 negative disease). Presence of metastatic breast cancer was ascertained on the basis of two or more healthcare encounters with a diagnosis of breast cancer (ICD-9-CM 174.x), plus two or more encounters with a diagnosis of distant secondary malignant neoplasm (196.2, 196.5, 196.8, 197.X-199.0), between January 1, 2000 and December 31, 2006 [16,17]. Receipt of chemotherapy was ascertained beginning 45 days prior to first diagnosis of secondary malignant neoplasm (through the end of follow-up), and was based on the presence of medical claims with a HCPCS code for a chemotherapy drug or a HCPCS, ICD-9-CM, or UB-92 code for administration of chemotherapy. A 45-day window was employed to capture instances where chemotherapy might have been initiated prior to first notation of metastatic disease on a health insurance claim. Date of initial receipt of chemotherapy was designated the "index date".

Patients with two or more medical encounters with a diagnosis of another malignant neoplasm (ICD-9-CM 140-172, 175-195, 200-208) 61 days or more before their index date were excluded from the study population, unless the site of the other neoplasm and the site of metastases was the same (e.g., malignant neoplasm of liver [155.0] and metastasis to liver [197.7]). Patients who were not continuously eligible for comprehensive health benefits during the six-month period preceding their index date were dropped from the study sample to ensure completeness in case ascertainment.

### Follow-Up

Follow-up began on the index date and ended with disenrollment from the health plan (in most instances, presumably due to death) or the end of the study period, whichever occurred first.

### Measures

Healthcare utilization was assessed by component of care, including inpatient services, outpatient care, and outpatient pharmacotherapy (i.e., drugs administered in an outpatient setting or dispensed at a retail pharmacy). Healthcare costs were estimated using paid (i.e., reimbursed) amounts, and were similarly characterized by component of care as well as on an overall basis. Analyses of outpatient services were further stratified by setting of care (emergency room, physician office, hospital outpatient, home health/hospice/skilled nursing facility, and other), and by type of service (e.g., evaluation and management, laboratory, radiology diagnostic, etc.) within selected settings, as feasible. Utilization and costs of pharmacotherapy were tallied on an overall basis and by selected medication groups.

### Analyses

Characteristics of study subjects were examined, including age, geographic region, payer, and prevalence of selected comorbidities. Age, geographic region of residence, and payer type were ascertained as of the index date. Comorbidities were ascertained based on the presence of diagnoses during the six-month pre-index period.

Cumulative total healthcare utilization and costs were estimated for each patient on a daily basis from index date through the end of follow up. Mean levels of utilization and cost of care were examined using Kaplan-Meier Sample Average (KMSA) methods. Using this technique, the follow-up period for each patient was partitioned into one-month intervals. Kaplan-Meier estimates of the probability of survival and continued health plan enrollment to the beginning of each interval were calculated. Expected utilization and associated costs of care were then calculated as the sum of the Kaplan-Meier estimates of the probability of survival to the beginning of each interval multiplied by corresponding estimates of utilization and costs respectively during the interval conditional on survival to the beginning of the interval [18]. Survival probabilities were calculated using dates of disenrollment, which was assumed to occur as a result of death; subjects who were observed through the end of the study period (i.e., December 31, 2006) were censored as of this date. Ninety-five percent confidence intervals (95% CIs) for costs were calculated using nonparametric bootstrapping [19]. Significance testing was not performed, as there were no a priori hypotheses. Cumulative component-specific healthcare costs, and the distribution of total healthcare costs by component, were estimated stratified by total cumulative cost (i.e., USD < 25,000, 25,000-< 50,000, 50,000-< 75,000, 75,000-< 100,000, 100,000-< 200,000, and ≥200,000, respectively). These analyses included only patients with complete cost data (i.e., those not censored due to the end of the study period, and thus for whom claims data were available from date of chemotherapy initiation through health plan disenrollment), as patients who were censored at the end of the study period undoubtedly incurred costs subsequent to this date.

## Results

### Patient Characteristics

The study population consisted of 1444 women; numbers excluded due to failure to meet various study entry criteria are provided in the Appendix (Additional File 1). Mean (SD) age was 59.1 (12.1) years; two-thirds of patients were older than 55 years (Table 1). Mean (SD) duration of follow-up was 532 (495) days (median = 366 days).

**Table 1:** Demographic and clinical characteristics of study subjects with metastatic breast cancer receiving chemotherapy

Parameter	Value	
	n = 1444	
Age (n, %)		
18-34	20	1.4
35-44	139	9.6
45-54	358	24.8
55-64	473	32.8
≥65	453	31.4
		
Geographic region (n, %)		
Northeast	176	12.2
Northcentral	399	27.6
South	784	54.3
West	85	5.9
		
Payer type (n, %)		
HMO	56	3.9
Indemnity	524	36.3
PPO	459	31.8
POS	393	27.2
Other	12	0.8
		
Comborbidities (n,%)		
Cerebrovascular disease	77	5.3
Coronary heart disease	152	10.5
Heart failure	26	1.8
Peripheral arterial disease	22	1.5
Diabetes	241	16.7
Kidney disease	29	2.0
Liver disease	100	6.9
Respiratory disease	547	37.9
None of above	634	43.9

### Healthcare Utilization

Sixty-four percent of patients were hospitalized at least once during follow up; the average number of hospital admissions was 1.7 per patient, and the mean number of hospital days was 10.7 (Table 2). Over the entire duration of follow-up, patients also averaged 58.9 physician office visits, 1.6 emergency room visits, 24.7 hospital outpatient visits, 5.5 home health/hospice/SNF visits, and 12.5 other healthcare encounters. Use of outpatient services was highest for radiology diagnostic services (89%), followed by laboratory (85%), procedures typically requiring anesthesia and/or sedation (69%), supplies (58%), and nuclear medicine (49%) services. Patients averaged 36.9 prescriptions during follow-up. Use of pharmacotherapy was highest for the combined category of analgesics, sedatives, and antidepressants (82%), followed by anti-emetics (81%) and anti-infectives (72%). Less than one-half of study subjects received cardiovascular agents (45%), erythropoietin stimulating agents (44%), gastrointestinal drugs other than anti-emetics (47%), fluids and electrolytes (45%), bisphosphonates (33%), and colony stimulating factors (33%).

**Table 2:** Healthcare utilization among patients with metastatic breast cancer receiving chemotherapy

	Mean
	n = 1444
Inpatient	
Acute hospital	
Admissions (#)	1.7
Days (#)	10.7
Outpatient Services	
Physician Office (#)	58.9
Emergency Room (#)	1.6
Hospital Outpatient (#)	24.7
Home Health (#)	5.5
Other (#)	12.5
Pharmacy Prescriptions (#)	36.9

### Healthcare Costs

**Figure 1:**
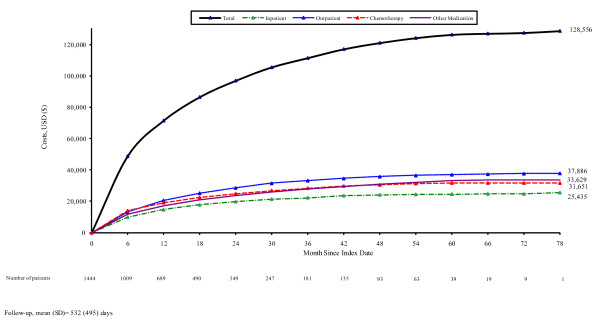
**Cumulative costs among patients with metastatic breast cancer receiving chemotherapy**.

Mean (95% CI) cumulative healthcare costs averaged $128,556 ($118,409, $137,644) from index date to the end of follow-up (Figure 1). Outpatient services accounted for 29% of total costs, followed by medication other than chemotherapy (26%), chemotherapy (25%), and inpatient care (20%) (Table 3). Among outpatient services, costs were highest for diagnostic radiology (19%) and radiation therapy (13%). Chemotherapy accounted for one-half of all medication costs, followed by colony stimulating factors (9%), erythropoietin stimulating agents (8%), bisphosphonates (4%), anti-emetics (4%), and pain, sedatives, and antidepressants (3%), among others. Chemotherapy was most often administered in physician offices (63%) and hospital outpatient departments (20%).

**Table 3:** Cumulative cost of medical-care services among patients with metastatic breast cancer receiving chemotherapy

	Mean (USD)(%)	
	n = 1444	
Inpatient		
Acute hospital	25,435	19.8%
Outpatient Services*		
Emergency Room	736	0.6%
Physician Office/Hospital Outpatient		
Evaluation and Management	2,851	2.2%
Laboratory	1,755	1.4%
Radiology Diagnostic	7,184	5.6%
Radiology Therapeutic	5,002	3.9%
Nuclear Medicine	891	0.7%
Procedures Requiring Anesthesia/Sedation	2,274	1.8%
Blood & Transfusion	176	0.1%
Physical & Occupational Therapy	318	0.2%
Medical & Surgical Supplies	841	0.7%
Mental health-care	58	0.0%
Other	10,362	8.1%
Subtotal	31,713	24.7%
Home Health/Hospice/Skilled Nursing	1,528	1.2%
Other	3,929	3.1%
Total	37,886	29.5%
Medication		
Chemotherapy	31,651	24.6%
G-CSF	5,018	3.9%
ESAs	5,644	4.4%
Pain, sedatives, antidepressants	1,821	1.4%
Anti-infectives	332	0.3%
Anti-emetics	2,664	2.1%
Biphosphonates	2,842	2.2%
Gastrointestinal	538	0.4%
Electrolytes, Caloric, Water	67	0.1%
Cardiovascular	573	0.4%
Blood products & Anticoagulants	661	0.5%
Other	13,475	10.5%
Subtotal	65,260	50.8%
TOTAL	128,556	100.0%

*Other than medication administration

**Figure 2:**
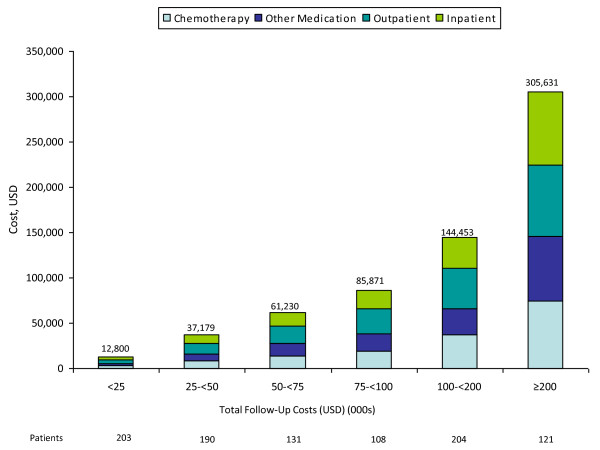
**Component costs of care among patients with metastatic breast cancer, by total cost**.

**Figure 3:**
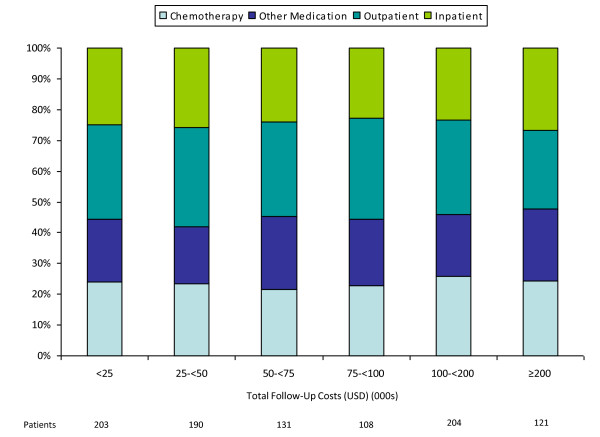
**Distribution of total costs of care among patients with metastatic breast cancer, by total cost**.

Among patients with noncensored cost data (n = 957), most (66%) had total costs less than $100,000 (Figures 2 and 3); outpatient and inpatient services accounted for 56% of total costs, while chemotherapy accounted for 23%. Among patients with total costs of $100,000 or more (34% of 957), corresponding figures were 53% and 25%.

## Discussion

Using a large health insurance claims database, we examined healthcare utilization and costs among patients with metastatic breast cancer receiving chemotherapy as their principal treatment modality (i.e., with no evidence of receipt of concomitant hormonal therapies or receipt of trastuzumab at anytime). Over a mean duration of follow-up of about 18 months, total medical-care costs averaged $128,556 per patient. Outpatient services accounted for 29% of total costs, followed by medication unrelated to chemotherapy (26%), chemotherapy (25%), and inpatient care (20%). Use of colony stimulating factors, erythropoietin stimulating agents, bisphosphonates, anti-emetics, and the combined category of analgesics, sedatives and antidepressants accounted for most medication costs unrelated to chemotherapy. In the outpatient setting, following services for evaluation and management, the largest cost drivers were associated with diagnostic imaging and radiation therapy.

Previous studies have examined economic costs in patients with metastatic breast cancer; from diagnosis to death, total costs have been reported to range from $41,590 to $82,973 (adjusted to 2005 US dollars) [16,17,20,21]. In prior studies, hospitalization has been reported to be the largest component of total costs, ranging from 33%-52% [16,17]. In our study, outpatient care represented the largest component of total costs. We urge caution, however, in comparing our findings with those reported by others, due to differences in study populations and methods. Of particular importance, our study is distinguished by the fact that we focused on patients for whom chemotherapy was the mainstay of treatment, and examined costs from initiation of chemotherapy (rather than diagnosis, as is typically the case in other studies) to end of follow-up. Other considerations include differences in expected survival across patient subgroups, operational definitions used to identify patients with metastatic disease, sources of utilization and cost data (e.g., primary, secondary, expert opinion), and how costs were measured (total vs attributable only), among others.

As with any retrospective database analysis, our study has limitations that should be considered when interpreting the results. For one, we used diagnosis and procedure codes from healthcare claims to identify women with metastatic breast cancer who were receiving chemotherapy. The accuracy of our case-finding methods is in fact unknown. We note that in a validation study that attempted to use ICD-9-CM diagnosis codes from Medicare claims data to identify patients with distant metastatic disease, the sensitivity and positive predictive value of such an approach were only 60% and 58%, respectively, when compared against information from the SEER program [22]. We suspect, however, that the positive predictive value of our methods was high (i.e., Type 1 error rate was low), as we identified study subjects on the basis of both diagnosis codes and evidence of receipt of chemotherapy. Sensitivity, however, could have been lower for a variety of reasons, including failure to use appropriate diagnosis codes for metastatic disease when metastases were indeed present, and generation of claims for biopsies and/or procedures prior to final pathological confirmation of clinical stage.

Second, while we sought to limit our attention to patients with HER-2 negative disease, due to limitations in study data, we had to infer HER-2 status based on absence of evidence of receipt of trastuzumab. While there might have been some HER-2 positive patients in the study sample who had contraindications to or refused trastuzumab therapy, we believe that most patients with known HER2-positive disease would have received trastuzumab, and therefore that exclusion of patients with evidence of such therapy yielded a population of patients with HER-2 negative disease.

Third, we may have failed to identify some patients who actually received chemotherapy, as administration of these agents is often billed using codes that lack specificity. Also, it typically takes one year or more for new products to receive a specific HCPCS code that can be used to identify their use in healthcare claims databases. During the intervening period, providers typically use nonspecific (or miscellaneous) codes that may also be used for other therapies.

Finally, due to HIPPA, vital status is not available in most healthcare claims databases. We therefore used health plan disenrollment as a proxy for death, as we believe that few patients with metastatic breast cancer are likely to change health plans, due to frequent limitations on coverage of pre-existing conditions. We therefore did not treat health plan disenrollment as a "censoring" event. If this assumption is incorrect, then our cost estimates may be downwardly biased. We note, however, that the median duration of follow-up in our study (~12 months) is similar to reported median survival (~9 months) in MBC patients with triple-negative (i.e., HER2-negative, hormone-receptor negative, progesterone-receptor negative) disease, not all of whom received chemotherapy [23].

## Conclusions

In summary, our findings suggest that healthcare costs among women with metastatic breast cancer who are receiving chemotherapy are substantial, especially in the outpatient setting. We believe that our study provides important additional information on the economic burden of metastatic breast cancer in a subset of patients for whom treatment options other than chemotherapy are limited.

## Declaration of Competing Interests

Gerry Oster, Angie Qin, Montserrat Vera-Llonch, and Derek Weycker are employees of PAI, an independent contract research organization that received financial support for this study from Amgen Inc. Andrew Glass is a practicing oncologist at The Center for Health Research, Kaiser Permanente Northwest, Portland, OR, and received funding for this research. Sue Gao is an employee of Amgen and holds Amgen stock. Rohit Borker was an employee of Amgen at the time of the study and holds Amgen stock.

## Authors' contributions

All authors made substantial contributions to the study in conceptualization and/or design of study, analysis and/or interpretation of data and manuscript preparation and/or review. MV, DW, AG, RB, SG and GO were extensively involved in study design and data interpretation. AQ's primary role was in statistical analyses. All authors read, edited and approved the final manuscript.

## Pre-publication history

The pre-publication history for this paper can be accessed here:

http://www.biomedcentral.com/1471-2407/11/250/prepub

## Supplementary Material

Additional File 1 **Appendix**. Click here for file
